# Metal Oxide Gas Sensors, a Survey of Selectivity Issues Addressed at the SENSOR Lab, Brescia (Italy)

**DOI:** 10.3390/s17040714

**Published:** 2017-03-29

**Authors:** Andrea Ponzoni, Camilla Baratto, Nicola Cattabiani, Matteo Falasconi, Vardan Galstyan, Estefania Nunez-Carmona, Federica Rigoni, Veronica Sberveglieri, Giulia Zambotti, Dario Zappa

**Affiliations:** 1 Consiglio Nazionale delle Ricerche (CNR), Istituto Nazionale di Ottica (INO), Unità di Brescia SENSOR Lab, Via Branze 45, 25123 Brescia, Italy; camilla.baratto@ino.it (C.B.); n.cattabiani@unibs.it (N.C.); matteo.falasconi@unibs.it (M.F.); vardan.galstyan@unibs.it (V.G.); e.nunezcarmona@unibs.it (E.N.-C.); federica.rigoni@unibs.it (F.R.); g.zambotti@unibs.it (G.Z.); dario.zappa@ino.it (D.Z.); 2 Dipartimento di Ingegneria dell’Informazione, Università degli Studi di Brescia, SENSOR Lab, Via Valotti 9, 25133 Brescia, Italy; 3 Consiglio Nazionale delle Ricerche (CNR), Istituto di Bioscienze e Biorisorse (IBBR), Via Madonna del Piano, 10, 50019 Sesto Fiorentino (FI), Italy; veronica.sberveglieri@ibbr.cnr.it

**Keywords:** metal oxides, nanowires, nanotubes, gas-sensors, photoluminescence, magneto-optical Kerr effect, surface ionization, electronic-nose, skin microbiota, *Enterobacter hormaechei*

## Abstract

This work reports the recent results achieved at the SENSOR Lab, Brescia (Italy) to address the selectivity of metal oxide based gas sensors. In particular, two main strategies are being developed for this purpose: (i) investigating different sensing mechanisms featuring different response spectra that may be potentially integrated in a single device; (ii) exploiting the electronic nose (EN) approach. The former has been addressed only recently and activities are mainly focused on determining the most suitable configuration and measurements to exploit the novel mechanism. Devices suitable to exploit optical (photoluminescence), magnetic (magneto-optical Kerr effect) and surface ionization in addition to the traditional chemiresistor device are here discussed together with the sensing performance measured so far. The electronic nose is a much more consolidated technology, and results are shown concerning its suitability to respond to industrial and societal needs in the fields of food quality control and detection of microbial activity in human sweat.

## 1. Introductions

Metal oxide semiconductors are widely studied and exploited layers in gas-sensing devices, mainly as conductometric sensors (or chemiresistors) i.e., transducing the reaction with the gaseous molecules through a change of the electrical resistance. The potential of chemiresistors arises from their sensitivity to several gases, their reduced size and weight, which make them suitable to develop portable instrumentation, the reduced preparation costs and the compatibility with Si technology [1].

On the other hand, these devices feature poor selectivity, which has stimulated researchers to look for different strategies to overcome this drawback. The most popular solution is to use the so- called electronic nose (EN), an array composed of different chemiresistors, each one showing a different response spectrum to gases, handled by a pattern recognition software [2]. Beside this, other approaches based on temperature profile protocols or the exploitation of different sensing and transduction mechanisms have been and are still being studied worldwide [3,4]. In this framework, the present paper reviews recent results achieved at the SENSOR Laboratory in Brescia (Italy) to address selectivity.

Devices based on electrical (chemiresistor), surface ionization, optical (photoluminescence) and magnetic (magneto-optical Kerr effect) transduction mechanisms are exposed in Section 2 as components of an ambitious goal, still to be reached, aiming to integrate these results into a single, multi-parametric device exploiting different signals from the same material to pursue a selective sensing.

The electronic nose technology is much more developed and prototypes have already been developed. Its working mechanism is briefly reviewed in Section 3 introducing recent models developed at SENSOR lab. Some key-applications are reported to show the effectiveness of this instrument to respond to the need of fields such as food quality control, where the complex composition of the head-space, often composed by hundreds if not thousands of compounds, makes the use of traditional analytical techniques extremely challenging.

## 2. Gas-Sensing Mechanisms

### 2.1. Chemiresistors

**Figure 1:**
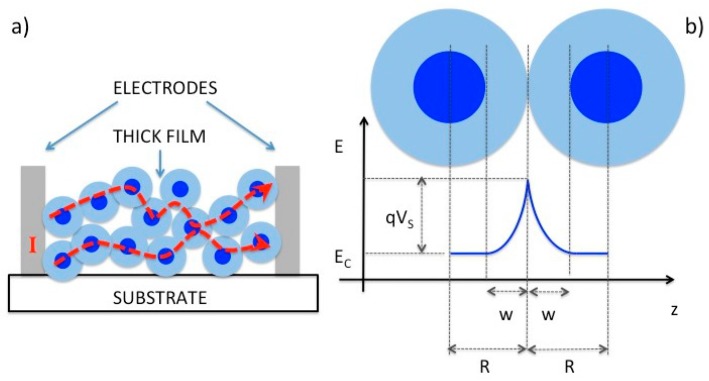
Schematic representation of the structure (**a**) and working principle (**b**) of thick film gas sensors. Reprint from [7].

The most widely used type of gas sensors is the chemiresistor based on n-type metal oxide nanostructures [5]. In such a device, the metal oxide layer is a sensing body which is a porous assembly of tiny grains (Figure 1a) and its gas sensing mechanism may be briefly described as follows: under exposure to air, oxygen is adsorbed on the grains of the material. The chemisorption of oxygen from the gas phase creates extrinsic surface acceptor states (O_2_^−^, O^2−^ and O^−^) immobilizing the conduction band electrons from the near surface region of the n-type semiconductor [1]. Thus, a depletion layer is created at the material interface due to the adsorbed oxygen from air under the ambient conditions (Figure 1b). The thickness of the depletion layer (*W*) is described in Equation (1), where *ε_r_* is the relative permittivity of the metal oxide, *ε*_0_ the dielectric constant of vacuum, *V_S_* the band bending, *q* is the electron charge, and *n_b_* the charge carrier density: The presence of other reducing or oxidizing gases may affect the density of charge carriers in the near-surface region of nanostructure [6]. The reducing gases will extract surface-bound oxygen atoms acting as donors for the n-type metal oxide. In contrast, oxidizing gases will immobilize further conduction-band electrons from the near-surface region by creating additional surface-acceptor states. The thickness of the depletion layer (Figure 1b) is changed due to the above mentioned oxidation-reduction reactions. Consequently, the presence of reducing gases increases the conductivity of the material, while the opposite is observed for oxidizing gases. The barrier height (*qV_S_*) created at the grain boundaries for the one-dimensional geometry depends on the density of surface states *N_S_* modulated by the oxidation-reduction reactions as expressed in Equation (2):

(1) $$W=\sqrt{\frac{\varepsilon_{r}\varepsilon_{0}V_{S}}{qn_{b}}}$$

The presence of other reducing or oxidizing gases may affect the density of charge carriers in the near-surface region of nanostructure. The reducing gases will extract surface-bound oxygen atoms acting as donors for the n-type metal oxide. In contrast, oxidizing gases will immobilize further conduction-band electrons from the near-surface region by creating additional surface-acceptor states. The thickness of the depletion layer (Figure 1b) is changed due to the above mentioned oxidation-reduction reactions. Consequently, the presence of reducing gases increases the conductivity of the material, while the opposite is observed for oxidizing gases. The barrier height (*qV_S_*) created at the grain boundaries for the one-dimensional geometry depends on the density of surface states *N_S_* modulated by the oxidation-reduction reactions as expressed in Equation (2):

(2) $$qV_{S}=\frac{q^{2}N_{S}^{2}}{2\varepsilon_{r}\varepsilon_{0}n_{b}}$$

This conductivity change mechanism is the basis of the chemical sensors’ working principles.

In addition, the physisorption effect of the gaseous spaces onto the surface of nanomaterials is another important issue for the fabrication of sensing devices. In this case the gas molecules within Van der Waals force react with the nanostructure and the electronic properties (current–voltage characteristics) of the material are changed [8,9,10].

Recent studies have demonstrated that the physisorption effect can also be used to improve the selectivity of the sensor devices [11,12]. The proper choice of the metal oxide, the capability to control the physical-chemical properties of these materials, i.e., their stoichiometry, porosity, grain size and shape, as well as the proper addition of dopants or surface catalysts are all effective routes adopted to tune the sensing properties of the obtained materials to provide different response spectra suitable for exploitation in an electronic nose instrument (see Section 3 for details). The work carried out world-wide in this framework has been well documented in several books and review papers, see for example [13,14,15].

In the present work, we focus in particular on quasi one-dimensional (1D) metal oxide nanostructures, which gained great attention due to their potentialities for applications in sensing devices [16,17,18,19,20].

A remarkable example of this strategy is given by titania nanotubes, for which the large surface area and fast charge transfer along single direction are very promising and well-controllable structures for the application in gas sensors [16,21,22,23,24,25].

In addition, the doping and functionalization of titania nanotubes with the particular materials may enhance their affinity and reactivity to certain gases [26,27,28,29]. Thus, varying the composition of tubular structures will be possible to improve their sensitivity and selectivity to different gases and to produce various specific sensors according to target gases and applications.

**Figure 2:**
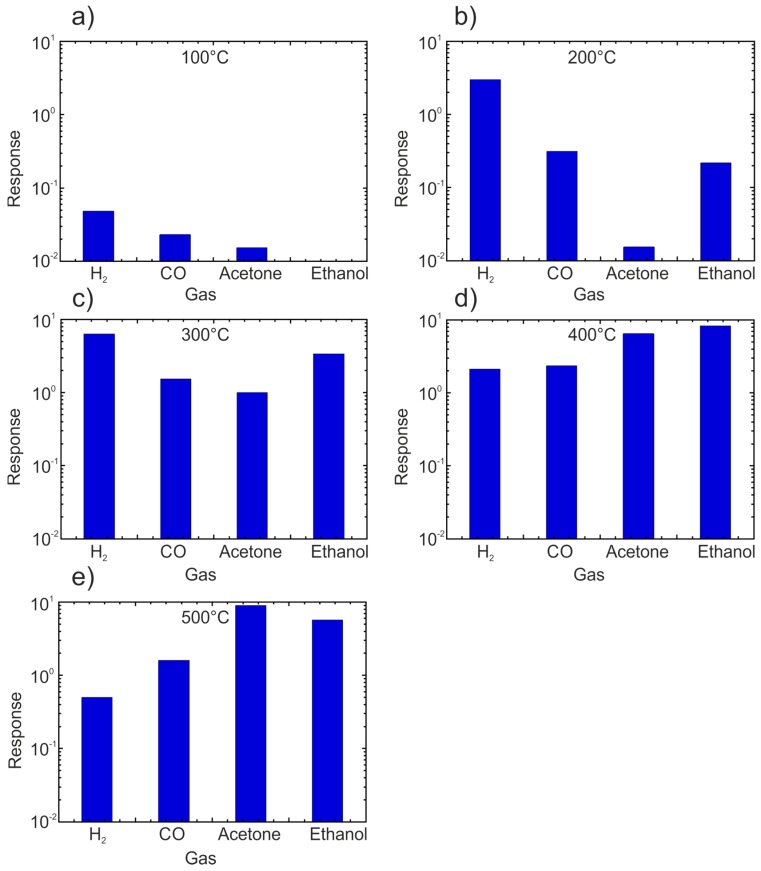
Response towards 500 ppm of H_2_, 500 ppm of CO, 50 ppm of acetone and 50 ppm of ethanol at the operating temperature of 100 °C (**a**), 200 °C (**b**), 300 °C (**c**), 400 °C (**d**), 500 °C (**e**), with 40% RH at the environmental temperature of 20 °C. Reprint from [32]. Response is calculated as the non-dimensional (G_gas_ – G_air_)/G_air_ where G_gas_ and G_air_ are the steady state values measured in the presence of the target gas (H_2_, CO, acetone, ethanol) and in the background air respectively.

**Figure 3:**
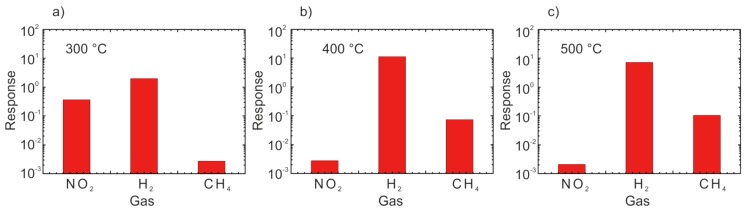
Response towards nitrogen dioxide, hydrogen and methane at 1 ppm, 1000 ppm, 50 ppm respectively and working the temperature of 300 °C (**a**), 400 °C (**b**), 500 °C (**c**) and RH 40% at the environmental temperature of 20 °C. Reprint from [38], copyright (2015), with permission from Elsevier.

We have developed an easy and cost-effective method for the fabrication of doped and mixed titania nanotubes [27]. Highly ordered and well-aligned nanotubular arrays have been obtained by means of electrochemical anodization of Ti and Nb–Ti metallic films. Briefly, thin films of metallic Ti and Nb–Ti were deposited on alumina substrates by means of RF magnetron sputtering. Then, the metallic thin films were anodized in two-electrode system at room temperature. The dimensions (diameter and the length) and the structure of the nanotubes is possible to tune by the variation of the anodization parameters and the treatment conditions [30]. Our studies showed that the tube dimensions have crucial effect on their sensing performance [31,32]. Meanwhile, the tuning of the structure crystallinity by variation of tubes’ thermal treatment regimes improves its stability at relatively high operating temperatures (Figure 2). We find out that the presence of Nb substitutional ions in the anatase TiO_2_ nanotubes hinders the anatase-to-rutile phase transition of the structure and increases their conductivity [32]. In the meantime, in Figure 2 clearly shows that the sensing performance (including the selectivity) of the nanostructures is affected by the operating temperature of the obtained structures. Generally speaking, since reducing gases reacts with chemisorbed oxygen, the responses to these kind of gases is often optimized at temperatures above 200–250 °C, at which oxygen starts to be activated [33]. Additional phenomena affecting the response amplitude are the activation of the reaction between the adsorbed gases and oxygen ions (thermally activated) and the Langmuir adsorption isobar (which decreases with increasing temperature) [34]. The low response to reducing gases observed at low temperature (100 °C) in Figure 2 is often observed with metal oxide chemiresistors working according to the chemisorption mechanism. Similarly, the response to oxidizing gases such as NO_2_ is often optimized at low temperature, since in this condition, the coverage of the metal oxide surface by oxygen ion is lower and then the material is more prone to oxidation (Figure 3) [35]. Beside these general and qualitative arguments, it’s worth noting that a more these parameters are strongly dependent on the material, its preparation, and the material—gaseous compounds considered and should be evaluated case by case. Indeed, comparing Figure 2 (TiO_2_) with Figure 3 (ZnO), it may be noted that the optimal temperature for H_2_ is different for the two materials: 300 °C and 400 °C respectively. Moreover, the addition of catalyst or the preparation of mixed oxides is a route often adopted both to increase the response to specific gases and to decrease the optimal temperature [36].

Another new type of nanostructure we have prepared combining the electrochemical anodization and the thermal decomposition methods [37]. We obtained ZnO nanoparticles connected to each other and forming chains with a few microns length [37]. Compared to other methods, the reported preparation process is quite simple and efficient: only thin films of metallic zinc were used, without any catalyst, additives, complex procedures or buffer layers. The structures demonstrated high response to NO_2_, H_2_, and CH_4_ (Figure 3). The response of the obtained structures strongly depends on its operating temperature, which is one of the principal parameter used to tune the response of chemiresistors for specific needs. The response to CH_4_ increases with the increase in working temperature, while the response to NO_2_ decreases as a function of the operating temperature. The prepared samples show an optimal response towards H_2_ at 400 °C [38]. Recently, we have demonstrated another strategy to control the chemical sensing performance of ZnO by coupling of graphene-based materials with the ZnO nanostructures [39]. The presence of reduced graphene oxide modified the response spectrum of the host ZnO by increasing the response amplitude to H_2_ and NO_2_ and reducing the response to CH_4_. The composite material was proposed as a good candidate for H_2_ detection with an optimal working temperature of 250 °C as shown in Figure 3.

The sensing mechanism described above is mainly suited in describing grain-like structures, it has been shown that using sensors based on materials with different morphologies is a suitable way to enhance the selectivity of a sensor array [40,41]. Considering nanostructures with different shapes, the sensing mechanism has to be tuned to take into account the different geometry.

Nowadays, research activities in the field of chemical sensing are focused not only on nanograins and related morphologies, but on single crystal nanowires too. Metal oxide nanowires are quasi-1D crystalline structures with diameters in the range of tens of nanometer, exhibiting properties that strongly differs from bulk material ones. They have a very high surface-to-volume ratio, meaning that a significant amount of material atoms are surface atoms. This enhances all surface phenomena, which are the key factors of all sensing mechanisms. Moreover, they usually are almost single crystals with fixed growth orientations, reducing instabilities due to percolation and hopping, and thus increasing the overall stability of the material, even at high temperatures required. Finally, particular effects like self-heating could be exploited only by fabricating nanowire-based devices. Indeed, thanks to the reduced mass and the absence of grain boundaries, the current flowing through a device composed by a single nanowire connected by two electrodes, which works as sensor signal, is enough to warm the nanowire by Joule effect up to the temperature required to activate the gas sensing reactions [42,43]. This technological solution allows for an extreme reduction of the power consumption, which may be reduced down to a few tens of μW [42], compared to the tens of mW required by micro hotplates [44]. Yet, due to the reduced mass of the single nanowire, self-heated nanowires show fast thermal kinetics (a few msec), comparable with those of micromachined substrates, that enable the effective exploitation of temperature profile protocols to further address selectivity. These protocols are based on a periodic modulation of the sensor temperature to induce a gas-dependent modulation of the sensor signal vs. time curve [3,45]. As an example, a square-wave voltage has been applied to a single nanowire device to extrapolate static (amplitude) and dynamic (time constant) parameters from the resulting periodic signal, which allowed to decouple the CO concentration from the humidity content in a CO-humidity mixture [46]. On the other hand, grain boundaries (or nanowire-nanowire interfaces) have been proposed by several authors as key elements to obtain high response amplitudes, in order to explain the response increase with increasing the average number of such interfaces in the sensitive layer of the chemiresistor devices [47,48].

**Figure 4:**
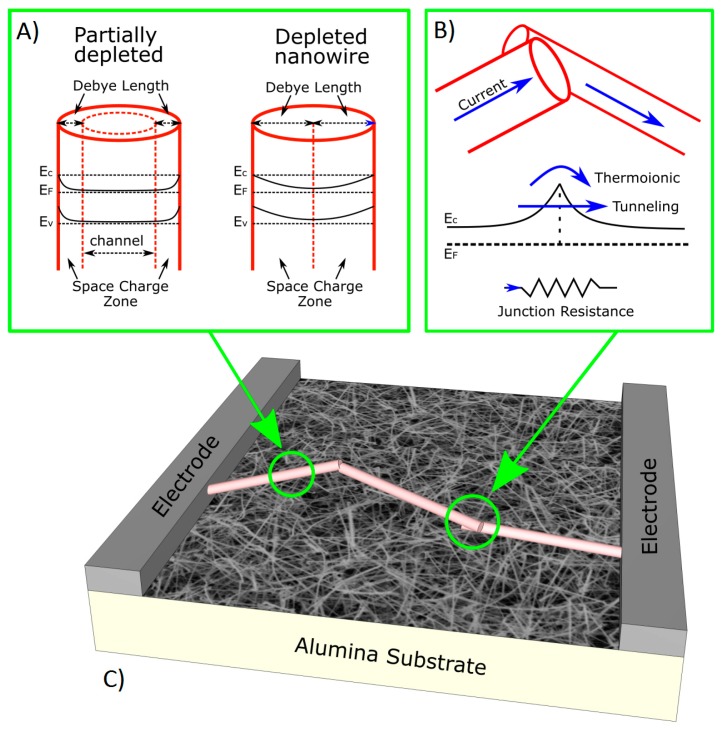
(**a**) Energy band diagram in a single partially-depleted nanowire and fully-depleted nanowire; (**b**) Energy band diagram at nanowire-nanowire junction, that could be modeled as a resistor; (**c**) Sketch of a conductometric mat-based device.

As for grain-like structures [49], adsorbed oxygen molecules (O_2_^−^, O^2−^ and O^−^) on the surface of metal oxide semiconductor disturb the free charge carrier distribution of the material. If we consider a single n-type MOX nanowire, like for example SnO_2_ or ZnO, these adsorbed molecules lead to the formation of a positively charged space charge zone, or depletion zone, forming a shell along the nanowire axis (Figure 4a, left). The width of this space charge zone can be expressed by the depletion layer width (W). If we consider a nanowire with diameter D, the width of the conduction channel (L_C_) in which carriers can move unaffected by surface charges become L_C_ = D − 2W. The depletion layer width is usually of the order of 10 nm, depending on the material, and thus comparable with the nanowire diameter. If the nanowire is very thin it becomes completely depleted (Figure 4a, right), and thus the electrical conduction is completely dominated by surface effects. Recently, the relation between the sensing performance and the size of single nanowire devices was also confirmed experimentally in literature [50].

In most conductometric sensing devices, however, a mat of nanowires instead of a single nanowire forms the sensing layer (Figure 4c). In these devices, nanowires are connected forming a dense network, and the junctions between nanowires play a strong role in the sensing mechanism. Due to the presence of a space charge zone on the surface, the junction could be modeled as Schottky barrier [51], in which thermionic emission and tunneling effects control the electrical current (Figure 4b). Reducing the nanowire diameter there is an increase of the space charge zones on the surface, and the Schottky barrier decreases. In mat-based devices, these junctions could be seen as resistors, and are more sensitive to the interaction with the surrounding atmosphere than the nanowire itself [51].

The reported sensing model is accurate; however, it does not take into account the catalytic properties of materials. In fact, as for other metal oxide morphologies, the sensing properties of nanowires strongly depends on the specific properties of the materials toward some chemical species. These properties are the key factors to enhance not only the sensitivity, but also the selectivity of devices in presence of different compounds.

In the past years, a strong research activity was performed at the SENSOR Lab to exploit the performances of nanowire-based devices. Highly crystalline nanowires of many metal oxides were synthetized directly on the sensor substrate, and the sensing performance were evaluated toward specific compounds of interest [52].

**Figure 5:**
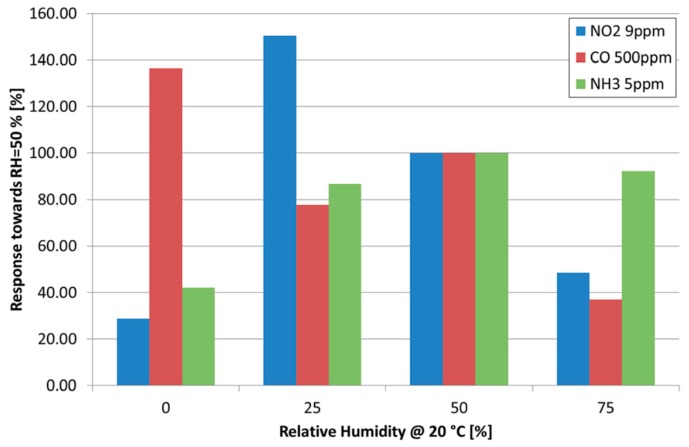
Influence of humidity on the 180 nm WO_3_ device response to nitrogen dioxide, carbon monoxide and ammonia. The y-axis reports the ratio between the response at the target value of relative humidity with respect to the reference value of RH = 50% at the environmental temperature of 20 °C. Sensor temperature is 200 °C. Reprinted from [56]—Reproduced by permission from The Royal Society of Chemistry.

Tungsten trioxide (WO_3_) is a widely studied material, thanks to its interesting sensing properties [53,54,55]. At the SENSOR Lab we used thermal oxidation in vacuum was used to synthetize very thin WO_3_ nanowires, with a diameter ranging from 15 to 40 nanometers showing an almost single crystalline structure [56]. Very good overall sensing performance were achieved, especially for hydrogen detection. We were able to integrate these nanostructures on commercial micro hotplates, proving that thermal oxidation is a very simple and scalable technique to produce commercial devices [57]. A comparison with other nanostructures such as nanofibers, showing a morphology composed by nanoparticles aggregated in elongated fibers, show that the nanofiber feature even higher response than nanowires (for example , the response against 1 ppm of acetone, is about 4 for the nanofiber layer [58] and around 2 for the nanowire layer [57]). This can be reasonably ascribed to the different structure of the two materials: nanofibers, with the inner structure composed by nanoparticles feature a large number of grain boundaries and a high porosity that is likely to optimize the response amplitude; nevertheless, issues related to grain coarsening phenomena affecting polycrystalline materials are expected to apply as well to these nanostructures, which are foreseen to show reduced stability in long time operation with respect to the nanowire based sensors. Moreover, we investigated the effect of a niobium addition to the tungsten layer [59]. The presence of niobium enhanced the response of tungsten oxide nanowires up to one order of magnitude. The effect of humidity on the response has also been studied, an example is shown in Figure 5. It is interesting to note that, as for all metal oxide based chemiresistors, the presence of water vapor in the atmosphere influence the response of WO_3_ devices. However, the effect is strongly dependent on which target chemical specie we are considering: when considering CO, the highest signal is obtained in dry air; when considering NH_3_ and NO_2_, instead, a small amount of humidity enhances the performances of fabricated sensors.

**Figure 6:**
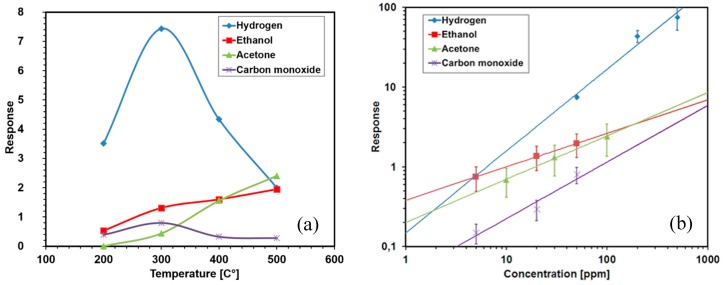
(**a**) Response of NiO nanowires towards some target gases; (**b**) Calibration curves for NiO sensor devices towards hydrogen at 300 °C, acetone and ethanol at 500 °C, and carbon monoxide at 300 °C. Reprint from [64] with permission.

In the field of chemical sensors, n-type semiconductor materials have always been more popular than p-type ones [60]. At the SENSOR Lab we also explored the properties of these poorly investigated metal oxide materials, like nickel oxide, for example. NiO is a p-type material that exhibit better overall performance compared to CuO, which is by far the most studied p-type material in literature [60,61,62,63]. For the first time NiO nanowires were synthetized by evaporation-condensation technique, and successfully integrated into a chemical sensing device [64]. These devices present very good sensing performance toward hydrogen: even if the maximum response is lower than one reported for WO_3_ nanowires, NiO is less sensitive to common interfering compounds like VOCs and CO (Figure 6).

Surface functionalization is another technique that could be used to enhance the selectivity of sensing materials for the detection of a target chemical specie. This is typically done dispersing catalytic nanoparticles (of Au, CuO, Ru, just to cite a few remarkable examples) over the metal oxide surface. In literature, several works are reported proving the effectiveness of surface functionalization by using heterogeneous catalyst [65,66,67]. For instance, Ru functionalization has proved to be a good candidate for the detection of NO_2_, while the addition of Cu strongly increases the performance toward H_2_S. For the detection of CO, Pd functionalization is worth to be investigated. However, a large potential is provided by organic receptors, which enable the exploitation of a variety of organic molecules. In this framework, we functionalized the surface of ZnO nanowires by using two different organic molecules, namely tris(hydroxymethyl)aminomethane (THMA) and dodecanethiol (DT), and we evaluated the improvement in the detection of NO_2_ gas. THMA-coated nanowires consistently displayed a small, enhanced response to NO_2_ compared to uncoated ZnO nanowire sensors [68].

### 2.2. Surface Ionization

**Figure 7:**
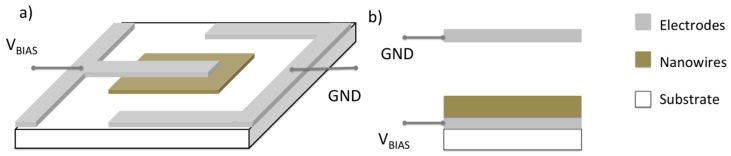
Schematic layout of the planar (**a**) and vertical (**b**) surface ionization device.

A different method to operate metal oxides for gas sensing is to heat up these materials at high temperature while applying at the same time an intense electric field up to induce the ionization of the adsorbed molecules. This sensing mechanism, referred to as surface ionization, has been recently exploited through a vertical layout (Figure 7) [69]. In these kind of devices the metal oxide layer is positively biased with respect to a suspended counter-electrode that collect the ionic current flowing from the metal oxide surface. The nanowire morphology has been shown to improve the ionic current, thanks to the capability of such morphology to enhance the local electric field, which, in turn, eases the ionization of the adsorbed molecules [69]. It’s remarkable the capability of these devices to work in air, at ambient pressure, using electrical fields of the order of 10^4^ V/cm, electrode separation of about 1 mm (which means a voltage of about 1000 V to have the required electrical field) and a temperature around 600–900 °C. The surface ionization mechanism is strongly depending on the properties of gaseous molecules such as their proton affinity and ionization energy, thus providing selectivity according to these molecular properties and a much reduced cross-sensitivity to humidity. The possibility to reduce humidity interfering effects directly by means of the sensor itself is a very important feature in view of the strong interfering effect felt by chemiresistor devices, for which, on the contrary, humidity effects have to be addressed through filters (subject to saturation and thus needing periodic replacement) or via software [70].

**Figure 8:**
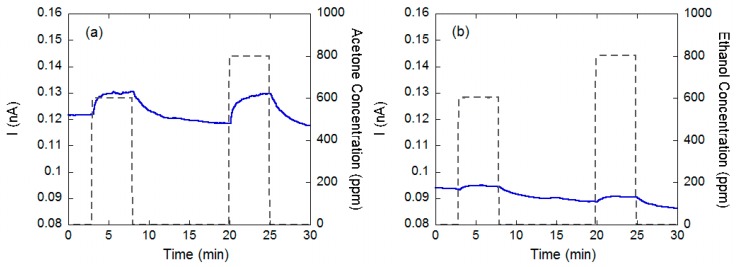
Dynamic response of a surface ionization device with planar layout, based on CuO nanowires to acetone (**a**) and ethanol (**b**).

An alternative approach to exploit this sensing mechanism is the planar layout, in which both the metal oxide and the counter-electrode lay on the same substrate (Figure 8). In this way, the shape and separation of anode and cathode can be precisely and easily controlled by means of lithographic techniques. A separation of about 200 μm can be obtained using the shadow mask method with the sputtering deposition technique. This method has been used to deposit both the Pt-counter-electrode and the metal oxide layer [71,72]. In particular a nanowire layer was grown starting from a metallic layer pattern by shadow mask and successively oxidized under controlled conditions to promote the formation of nanowire structures suitable for surface ionization [73].

The main difference between the vertical and the planar configuration is the temperature of the counter electrode, cold in the former configuration, warmed at the same temperature of the nanowire layer in the latter. As a consequence, devices prepared according to the vertical scheme feature a highly asymmetric current-voltage characteristic [69], while asymmetry is largely reduced with the planar scheme due to the capability of the warm Pt electrode to ionize adsorbed molecules. In this case, the difference is due to the nanowire morphology (if a nanowire layer is used) and the different affinities of Pt and the metal oxide with the adsorbed molecules [71].

Other worth to note important differences are the lower temperature and voltage that can be used to promote surface ionization phenomena in the planar configuration. Indeed, the planar configuration can be activated already at temperatures around 350 °C and voltages of 30 V (corresponding to an electric field around 1500 V/cm), while 600–800 °C and 10^4^ V/cm are necessary with the vertical layout [71]. An example of the response exhibited by a surface ionization device based on a CuO nanowire layer and planar configuration to acetone and ethanol is reported in Figure 8. These sensing measurements show a behavior coherent with the ionization energy argument (9.70 eV for acetone, 10.48 for ethanol [74]).

On the other hand, it’s worth to note that the dependence on ionization energy/proton affinity, which is at the basis of the enhanced selectivity of surface ionization based devices, is still present, but in a reduced manner, in the case of horizontal layout with respect to the vertical configuration [75]. These arguments make the planar configuration more sensitive than its vertical counterpart to humidity, whose high ionization energy.

Surface ionization devices based on a single nanowires have been realized as well, exploiting a planar layout and depositing a Pt counter-electrode by means of Focused Ion Beam (FIB) lithography [76]. The reduced gap between the nanowire and the counter-electrode, which can be controlled at the scale of a few hundreds of nm and the reduced diameter of the nanowire itself, allowed to obtain appreciable surface ionization current and sensing response working with voltage bias as low as a few volts and activation temperature lower than 300 °C.

### 2.3. Optical Sensors

An optical gas sensor is intended as a device that changes its optical properties due to the interaction with a certain gas. In particular, at the SENSOR Lab, we observe gas effects on the photoluminescence (PL) emission mainly on ZnO NWs and on SnO_2_ NWs [77,78]. SnO_2_ material has only defect emission, thus the PL signal is very much dependent on the preparation conditions, making it less interesting for sensing application than ZnO.

Zinc oxide is an important material for optoelectronics in view of its highly efficient light emission in the UV range, related to near band edge (NBE) excitonic emission. Most ZnO nanostructures also exhibit defect-related green luminescence, as clearly shown in the case of ZnO nanowires here studied. Also for ZnO, the visible emission is strongly dependent from preparation conditions.

In the literature we can find reports on optical sensors based on PL quenching of ZnO, starting from the first works of the SENSOR lab group [77,78,79,80], that was followed by the work of other groups [81]. TiO_2_ was also considered as optical sensors for O_2_ detection based on PL quenching [82,83].

**Figure 9:**
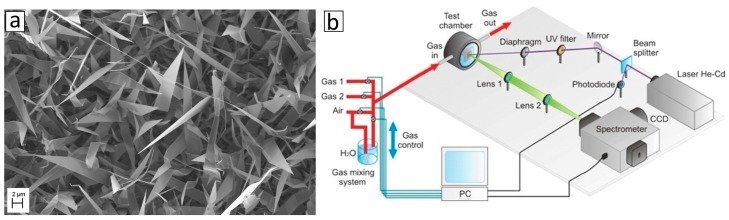
(**a**) SEM image of ZnO NWs; (**b**) Photoluminescence (PL) spectrum is acquired perpendicular to the sample surface by a single spectrograph and a CCD camera. The excitation wavelength is 325 nm (He-Cd laser). A sealed chamber is used for gas tests.

ZnO nanowires were prepared by evaporation condensation technique, as described elsewhere [80]. Figure 9a is a Scanning Electron Microscope (SEM) image of the ZnO NWs, showing that the sample is constituted by single crystalline nanowires that grows from the substrate in random directions, partially covered by some platelets of ZnO (that are also single crystal).

To perform optical characterization in presence of gas species, we have realized a macro-PL setup shown Figure 9b. The test chamber is made of stainless steel and equipped by quartz window. The sample works at room temperature or can be heated up to 400 °C (if needed). The synthetic air flux in the test chamber is maintained constant to 0.3 L·min^−1^, at 20 °C and atmospheric pressure, and mixed with the desired amount of gaseous target molecules. Photoluminescence (PL) measurements were carried out with a He–Cd excitation laser source (325 nm). PL spectra were acquired perpendicular to the sample surface using a single spectrograph and a Peltier cooled CCD camera.

In addition to the macro setup, at SENSOR Lab it is possible to carry out continuous-wave Raman and PL spectroscopy in a microscopic configuration by a Horiba modular confocal micro-Raman system, allowing the investigation and characterization of isolated ZnO micro and nanostructures.

**Figure 10:**
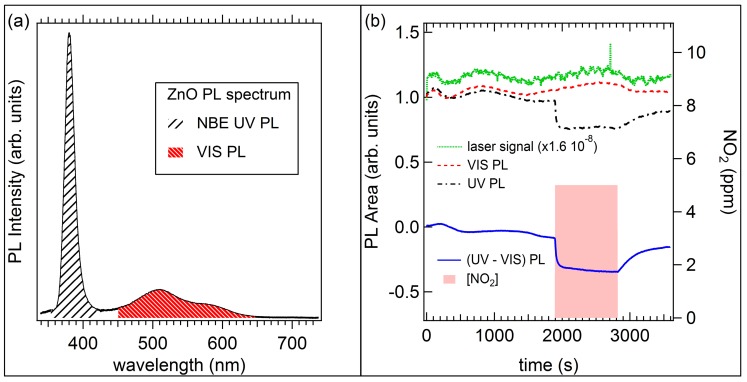
(**a**) Photoluminescence (PL) spectrum of ZnO NWs with ultraviolet Near Band Edge (NBE) and visible emission; (**b**) PL optical response to 5 ppm nitrogen dioxide gas.

Figure 10a shows the PL spectrum of ZnO NWs acquired in dry air before measurements: in accordance with results reported in literature, we observed the UV near band edge (NBE) exciton peak at 380 nm and a broad band emission in the visible range 450–650 nm. To examine the kinetics of the involved processes, PL data were taken every 5 s and area under NBE and visible peak was extracted as a feature for each sampling. In Figure 10b the PL optical response to 5 ppm nitrogen dioxide gas exposure is split in the two emission region: the PL Area under the NBE UV peak vs. time (black dash-dot line), the visible PL emission (red dashed line) and the laser signal as the power per unit area in arbitrary units (green dotted line) are shown. We can observe that the visible PL (VIS PL) time evolution reflects the laser signal (affected by short and long time instabilities), so we can conclude that no VIS PL variation is observed upon NO_2_ gas exposure. The UV PL shows, in addition to the variations due to the laser instability, a sensible variation due to NO_2_ gas interaction. To remove the long term laser induced fluctuations, the difference between the UV and VIS PL has been plotted versus time (blue solid line). This curve has been fitted with a double exponential function for the rise time, obtaining the following decay-time parameters τ_1_ = (18.4 ± 0.2) s, τ_2_ = (395 ± 12) s; and with a single exponential function for the recovery time: τ = (242 ± 1) s. The parameters that influences response and recovery times are currently under investigation. After NO_2_ introduction, quenching of the PL signal was observed, but no peak shift. The quenching was fully reversible as dry air is restored in the test chamber. Response time is of the order of tenths of seconds and recovery time of the order of hundreds of seconds.

In our previous work [77], we observed NO_2_ detection properties that were independent of the emission band selected for detection. The difference in sample preparation and morphology of ZnO samples analyzed here could be responsible of the modified behavior of PL signal, but careful investigation is in process. It is indeed of high relevance to have a signal linked only to NBE emission of ZnO which is much less dependent on the sample preparation conditions than defect band is.

The response to NO_2_ using an optical readout was also demonstrated for lower concentration (down to NO_2_ concentration of 0.1 ppm in air, which is the limit fixed by Italian legislation for alarm level for NO_2_ presence outdoor) [77].

Concerning the working mechanism for the optical gas response, the presence of nitrogen dioxide on the nanowire surface influences recombination processes, destroying radiative recombination paths. NO_2_ adsorbed over the surface as ${\mathrm{NO}}_{2}^{-}$ is responsible for the charge transfer between the gas molecule and the semiconductor surface that induced the electrical response. The optical response, much faster than the electrical one, allows us to attribute the PL quenching to NO_2_ physisorbed over the surface and to the creation of non-radiative recombination paths, as in the case of SnO_2_ [78].

Time-resolved photoluminescence measurements [77,79] showed that the observed small modifications of recombination rates due to introduction of NO_2_, are not proportional to simultaneous changes in photoluminescence intensity. Thus we can hypothesize a model in which the NO_2_ molecules act as static quenchers.

Two static quenching mechanism are possible: one is the suppression of radiative states due to chemisorption of NO_2_ molecules on the ZnO surface. The other one is the increase of space-charge region due to NO_2_ adsoprtion on surfaces with a consequent band bending: in this case part of the deep level states near to surfaces may lie below the Fermi level so that they become unoccupied and may no more recombine with holes giving rise to the luminescence.

To investigate the gas sensing selectivity, we studied the effect of interfering gases like ethanol and relative humidity that could give spurious signal. The results are reported in [77], Figure 4. A quenching of 8% was observed with 2 ppm of NO_2_, while the opposite effect was observed with ethanol and RH, which acted as PL enhancer. The relative response to ethanol concentration (1000 ppm) was 1.5%. The PL increase with RH gave relative response of 2.8%, 3.9% and 4.6% respectively to 20%, 50% and 70% RH, so this interfering effect must be taken in account when dealing with all-optical gas sensor. In another work [80], we also studied the effect of other gases on PL quenching, like ethanol, relative humidity and CO. No response was observed for CO, while a quenching of 4% was observed changing from dry air to 30% relative humidity and an increase of 7% was observed for 900 ppm of ethanol.

Therefore we can conclude that the optical sensor is almost insensitive to common interfering gases for NO_2_ detection, such as ethanol and methanol. This may partially arise from the low working temperature (below 150 °C) of such an optical sensor, while VOCs usually chemisorbs with MOX surface at much higher temperatures than NO_2_ (Section 2.1).

Overall, these results suggest that this approach is potentially suitable for the development of an optical gas sensor working at RT, that allows to avoid the low reliability electrical contacts on nanowires.

A low cost application of the optical sensor can be implemented by LED excitation (instead of laser excitation), thanks to the high emission efficiency of ZnO nanowires [77]. Moreover, the PL quenching (variation) can be detected simply by selecting the desired spectral area via a bandpass filter and a photomultiplier [84] (or even a photodiode).

### 2.4. Magnetic Sensors

The use of a different transduction mechanism for gas sensing is of utmost importance in order to obtain a new sensor that differs for some special characteristic, for example the species it can detect, or the working temperature. Metal oxide gas sensors are used since many decades to detect a gas species at high working temperature (250–500 °C) that is needed to promote gas reaction with the oxygen ionosorbed over the semiconductor, inducing a variation in the resistance of the material. As a matter of fact, high temperature operation could raise the problem of ignition of fuels when detecting explosive species. Hydrogen, for example, can explode when mixed with atmospheric oxygen at concentration of 4% (Lower Explosive Limit–LEL). Thus room temperature detection is very important.

Our idea is to exploit a new sensing mechanism that involves a magneto-optical readout of the signal, Co/ZnO hybrid device was thus prepared by RF sputtering and investigated using a magneto-optical Kerr effect (MOKE) magnetometer. Since MOKE is a surface sensitive technique, it is influenced by reaction taking place on the ZnO surface. This would pose the base for a future generation of magnetic sensing devices.

The device we developed differs from dilute magnetic semiconductors (DMS) that have been studied recently in literature, which are wide bad gap semiconductors doped with Fe, Co and Ni in order to behave as ferromagnets [85,86]. DMS needs very high magnetic field (order of KOe) in order to work as gas sensor, while in Co/ZnO hybrid device uses magnetic field in the Oe range.

The MOKE setup is known and applied for surface plasmon resonance (SPR) sensors, where the sensing mechanism relies on the on the registration of the resonance associated with the excitation of surface plasmon-polaritons which are electromagnetic waves propagating along the metal–dielectric interface [87,88].

**Figure 11:**
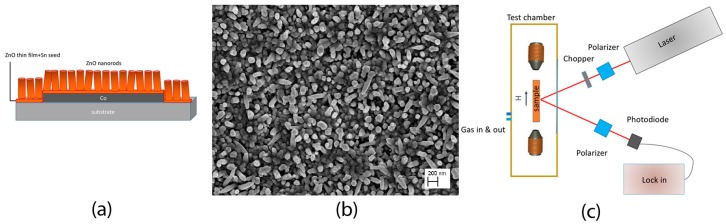
(**a**) Sketch of the sensing device; (**b**) SEM image of ZnO NRs surface; (**c**) Scheme of MOKE readout for gas sensing application.

We demonstrated that the hybrid Co/ZnO nanorods (NRs) could sense H_2_ and CO at room temperature [89,90]. The sensing device was entirely realized by RF sputtering, a scalable and low cost technique. A sketch of the device is shown in Figure 11a: First, a Co ferromagnetic layer of 150 nm was deposited on the ceramic substrate, covered by thin layer (50 nm) of ZnO that prevent oxidation and capped by ZnO nanorods (NRs) magnetoelectrically coupled at the interface. The ZnO NRs growth is promoted by non continuous layer of tin deposited at 400 °C as the seed for ZnO growth, followed by ZnO growth in inert atmosphere. After preparation the sample was annealed at 300 °C in air.

We have chosen ZnO as the material which interacts with the gas is ZnO, since it is a well know semiconductor in sensing field, and it can be grown in NRs shape to ensure high surface to volume ratio needed for high sensor response [80]. Because the underlying Co layer is very sensitive to oxidation, the ZnO nanowires grown by high temperature technique (like by Vapour Liquid Solid) would degrade the magnetic properties of the device. We thus modified the sputtering deposition of thin films from ZnO target in order to obtain highly porous surface constituted of nanorods. All deposition was carried out in vacuum, thus preventing Co oxidation. Figure 11b shows a SEM image of ZnO surface: polycrystalline nanorods grow mainly perpendicular to the sample surface with average diameter in the range 80–100 nm.

The MOKE setup used in the experiment is in longitudinal geometry, using an *s*-polarized He-Ne laser (λ = 633 nm). The light is linearly polarized and passes through an optical chopper which modulates its intensity at a frequency of 400 Hz. The reflected light passes through a second polarizer that is rotated close to the extinction in order to enhance the signal to noise ratio and to detect only the Kerr signal, recorded through a Si photodiode. The output is then acquired using a lock-in amplifier. The scheme of the setup is reported in Figure 11c. The sample is placed in a closed chamber with gas inlet and outlet and quartz window. A small electromagnet inside the chamber allows to apply a magnetic field. We have to stress that the magnetic field used in these measurements is very low (50 Oe maximum), especially if we compare it with the magnetic field (of the order of KOe) that is needed to measure gas sensing properties in diluted magnetic semiconductors [91].

**Figure 12:**
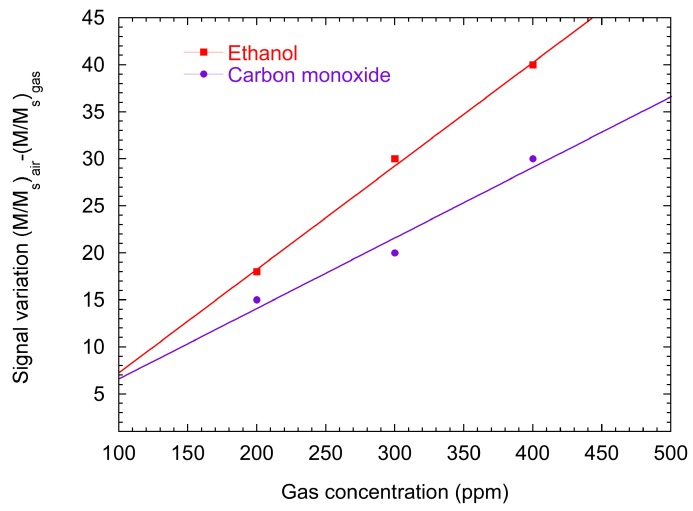
Sensor response of the magneto-optical gas sensor to H_2_ and CO concentration (200-300-400 ppm) in dry synthetic air. Measurements are carried out at room temperature.

The sensing measurements were acquired by measuring the changes in magnetization of the Co/ZnO hybrid with an in-plane magnetic field of 50 Oe. The changes in magnetization are induced by gas interaction. The gas carrier was dry synthetic air, and after a stabilization period H_2_ (200-300-400 ppm) and CO (200-300-400 ppm) were introduced. The interaction with reducing gases reactions induce a decrease in the magnetization proportional to the target gas concentration. The baseline signal is completely recovered after dry air restoration, with quite fast response and recovery times (both less than 60 s) [89]. The results are resumed in Figure 12: we plotted the sensor response R, defined as R = (M_air_ − M_gas_)/M_air_, where M_air_ (M_gas_) is the measured magnetization in air (gas) with the applied in-plane magnetic field (M) of 50 Oe. We can observe that response is linearly proportional to gas concentration, while in case of electrical gas sensing the dependence of relative response to the concentration is described by a power law [92]. To exclude that the observed sensing properties are due to changes in the reflectivity or in the optical properties of ZnO NRs, experiments at zero magnetic field were carried out, showing no magnetization variation upon gas interaction.

Tests done showed that the sensor signal is insensitive humidity variation from 0% to 50% RH at RT) [89]. Up to now only hypotheses were formulated on the mechanism responsible for decrease in magnetization observed in presence of reducing gases, taking into account that only a thin layer at the interface between ZnO and Co is Cobalt –doped Zn, while the Co is completely covered by ZnO preventing oxidation [90]. Due to room temperature operation we expect that H_2_ (CO) is physisorbed and interact with oxygen species that resides on ZnO NRs. An electron is released back by oxygen desorpting from the surface into the ZnO conduction band and thus is transferred to the Co layer. An increase of the Fermi energy of Co is expected owing to the dn electrons generated in ZnO and entering the Co [93].

As described above, ZnO nanowires or nanostructures can be employed to realize optical and magnetic gas sensors. This combined concept of sensors that exploit different gas sensing mechanisms and working conditions, could allow us to reach a deeper understanding of the metal oxide semiconductors gas sensing behavior.

**Figure 13:**
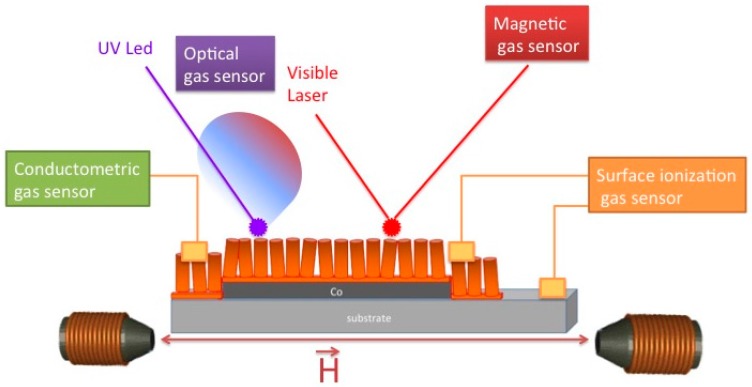
Scheme of multisensor chip for gas detection merging electrical, optical, magnetic, surface ionization sensing.

To take advantage of different sensing mechanism applied to the same semiconductor, it is possible to realize a multisensor chip for gas detection featuring electrical, optical, magneto-optical and surface ionization sensors. The proposed multisensor is schematized in Figure 13: two electrical contacts allows the detection of electrical resistance, while UV led excite the PL signal that can be acquired by a photodiode and filter. A visible laser and a MOKE setup allow the detection of the magnetic sensor signal. A gap without nanowires and another contact is thought for surface ionization measurements. Each sensor has its specific target molecule and operating temperature, providing increased selectivity.

## 3. Electronic Nose Applications

### 3.1. Electronic Nose Working Mechanism

An electronic nose is a chemical sensor device that uses an array of gas sensors with overlapping selectively along with a pattern recognition (PARC) software. ENs were developed to imitate human olfaction, i.e., smell the odor as a global fingerprint. The instrument traditionally consists of four main building blocks: the gas headspace (HS) sampling device, a chemical gas sensor array, an electronic board for signal acquisition and conditioning, and a PARC module.

Since the end of 1990s, various EN technologies have been developed and used at the SENSOR Lab. For many years the EOS series EN (EOS835, EOS507) produced by SACMI IMOLA S.C. (Imola, Italy) have been used in different application contexts, including the case studies that will be illustrated in the next sections. These systems have been described in previous papers [94,95,96,97,98,99,100].

Due to the sensitivity of metal oxides to humidity and considering that biological or food samples may often release different humidity content in the head-space, the EOS507 has been equipped by the manufacturer (SACMI IMOLA S.C., Imola, Italy) with a humidity compensator that allows to work at a constant humidity level. As better detailed in literature [98], the system filters the ambient air used as reference and humidify it to the level settled by the user. This enhances the performance of the EN [98] but at the cost of an increased system complexity.

**Figure 14:**
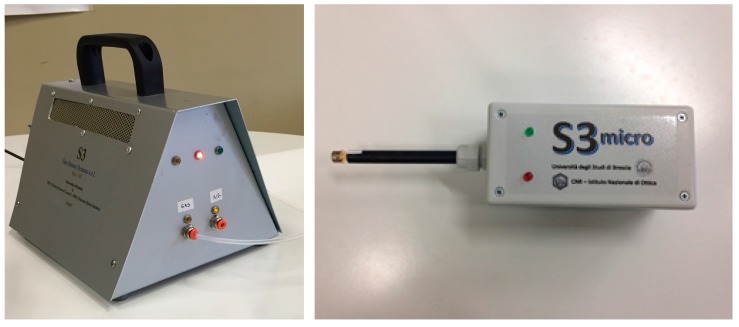
Images of S3-mini (**left**) and S3-micro (**right**) developed by SENSOR Lab (courtesy of NASYS SRL).

More recently, a new EN called Small Sensors System (S3) [101] that incorporates MOX-NWs gas sensors in the array was developed in cooperation with NASYS srl, a spin-off of the University of Brescia. Two prototypes are currently under development called S3-micro and S3-mini (Figure 14).

The S3 is a miniaturized portable, compact, and automatic instrument. It consists of a pneumatic assembly for dynamic sampling (pump, electro-valve, electronic flow meter), a miniaturized gas sensor chamber of 5 mL internal volume which hosts up to 10 sensors, a home-made electronic board for sensors control and signals acquisition, and a dedicated (cloud-based) software for data handling, visualization and training.

The sensing elements are zinc and tin oxide crystalline nanowires and more traditional thin film MOX sensors. Specifically, in current prototypes, there are six MOX gas sensors, three of them prepared by the nanowire technology and the others by the Rheotaxial Growth and Thermal Oxidation (RGTO) thin film technology. Out of the three nanowires in the array, ZnO and SnO_2_ sensors with different operating temperatures are used. Regarding RGTO sensors, the S3 uses different oxides such as SnO_2_ catalyzed with noble metals (Au, Ag, Mo, Pd and Pt) nanoparticles, WO_3_ or MoO_3_. All the sensors in the array are rather unspecific and specificity is provided by the combination of their responses.

The S3 supports dynamic or static headspace sampling unit (optionally with an autosampler HT200H, HTA srl, Brescia, Italy). Static headspace has clear advantages in terms of reproducibility and repeatability. The HS generation parameters (incubation temperature, time and so on) can be fully and accurately controlled. Besides, the HS analysis is carried out without perturbing the equilibrium conditions—this ensures there are no artefacts in the sensor response due to changes of HS concentration during the measurement. Finally, static headspace may be used to perform long runs of measurements, thus improving the training set collection and the device calibration. Nevertheless, the use of static headspace sampling strongly limits the EN sensitivity due to the small amount of available headspace (about 5 mL) and consequently low carrier flow rate values (10 mL/min). Therefore, in some applications, dynamic headspace is to be preferred; it basically consists of a pump and a flow controller that conveys the odor sample under investigation from a vessel (typically 100 mL in volume) into the sensor array chamber. Sensitivity can be enhanced by almost one order of magnitude and sensor recovery time can be correspondently halved by using dynamic headspace.

The S3 instrument remote control can be performed through wireless interfaces (Bluetooth) or Ethernet. The system is designed to be controlled by an app (Android) and gather high amount of data through a user-friendly interface. The data are elaborated off-line by graphic visualization tools by a home-made software package formerly based on MATLAB and now evolved into a Java web-app. The software includes univariate and multivariate statistical analysis functions among which Principal Component Analysis (PCA), allowing easy data manipulation (reshaping of matrices, data set fusion, relabeling) and plots customization. The systems also implements supervised classification tools, under development, by different PARC algorithms such as Support Vector Machines (SVM) and Artificial Neural Networks (ANN). A recent and comprehensive overview of PARC techniques for machine olfaction has been provided by De Vito et al. [102].

**Figure 15:**
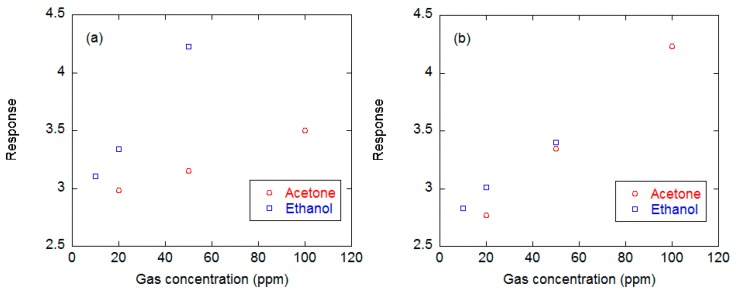
Calibration curves acquired with chemiresistors based on SnO_2_ RGTO thin film (**a**) and nanowire (**b**) mounted in the EN against ethanol and acetone.

Some selected applications in which the chemiresistor devices developed at SENSOR have been integrated in the aforementioned EN platforms are discussed in the following paragraphs. A key feature of the adopted sensor arrays is the integration of the nanowire and RGTO thin film technologies we did as we were progressing in the development of nanowire-based devices. Indeed, nanowires and thin films, even if based on the same material exhibit different response spectra. An example is shown in Figure 15, which reports the calibration curves of chemiresistors based on pure SnO_2_ films prepared by means of nanowire and RGTO technologies lodged in the EN. It is evident that the nanowire device is more sensitive to acetone than its thin film counterpart while the film device is more sensitive to ethanol. Such differences can be useful features in the EN to enhance its selectivity capability.

### 3.2. Monitoring of Human Microbiota

The primordial sense of smell will be essential for living, this sense is mediated by specialized sensory cells of the nasal cavity of vertebrates, which can be considered analogous to sensory cells of the antennae of invertebrates. In animals is essential as example, to find prey and food, to detect harmful substances in food (rotten food or putrescence), to mark territory, to detect warming signals.

In fact, animals and humans emit a complex array of non-volatile and volatile molecules, depending on: Stress condition (if humans are particularly scared or happy), DNA (gender or geographical conditions), usual diet (omnivore, vegetarian, vegan, etc.).

All these parameters and many other reported in literature [103,104], contribute to create a characteristic olfactory finger-print of every human, but at the same time with many common volatile bio-markers.

In fact, we could distinguish between factors more or less variables in time and less tied to our personal habits [105]. Some odors are stable over time (genetically based or they may vary with environmental or internal conditions (like pathology) [106].

Recently gas sensor devices as some kind electronic noses have been used in the monitoring of human behavior in many different aspects since safety to health monitoring. Regarding safety aspects a broad list of Volatile organic compounds VOCs have been identified in order to be used as tracking molecules to rescue people in natural or urban disaster as earthquakes [107]. The major advances regarding the identification of human volatile compounds have been done in medical environment. In this regarding remarkable advances have been done in the application of electronic devices for the rapid and noninvasive detection of respiratory diseases as infections [108,109], asthma [110] and lung cancer [111] and gastrointestinal track diseases as colorectal cancer [112,113].

Human skin is known to be colonized by a huge number of microorganism that live as commensals on the surface and within the follicles. Hundreds of different kinds of substances appear in human fingerprint, which can be classified into several groups according to their functional groups such as carboxylic acids, alcohols, aldehydes, aliphatic, esters, ketones, amines, heterocycles.

In this framework, at the SENSOR lab we recently started a research activity dedicated to study the suitability of an EN (the S3 device) to detect the activity of microbiota in sweat media [114].

Sweat is the natural environment of several microbiotas. Analysis of microbiota populations and their activity is useful for different applications, including early disease detection, health and safety monitoring. Moreover, the bacterial activity is responsible for the development of sweat odor, whose detection may be absolutely useful in safety and security applications such as rescue of people after natural disasters as earthquakes.

To prepare samples, in a first step the skin microbiota of three anonymous volunteers was sampled with a cotton swab and innoculated in a Petri dish containing Plate Count Agar Media (PCAM) and incubated for 48 h at 32 °C. PCAM is a microbial media for general microbial counts that also allows the growth of most microbial species.

For each cm^2^ of skin it’s possible to find 10^4^–10^5^ Colony Forming Unit (CFU) and an adult human being has 2 m^2^ of total skin surface, which means that overall it carries from 2 × 10^8^ − 2 × 10^9^ CFU. Besides an adult human being produce in normal conditions 400 mL of sweat per day [115]. So far, three different blends of microorganisms have been inoculated in artificial sweat in a concentration of 5 × 10^6^ CFU/mL in order to mimic the normal conditions of the skin.

The electronic nose was provided with the auto-sampler headspace system HT280 (HTA srl, Brescia, Italy), supporting a 40 loading sites carousel and a shaking oven to equilibrate the sample headspace. A volume of 2 mL for every dilution was placed individually in a sterilized chromatographic vial of 20 mL. Once inoculated all the vials were cover with an aluminum crimp, a coated PTFE/silicon septum and crimped. This operation was repeated during the 80 h of analysis (from T0 to T80). It was used synthetic chromatographic air with a continuous flow rate of 10 mL/min to perform the sensor baseline and the recovery time was 28 min. The used device in this work is the S3 (Small Sensor System). S3 is equipped with six MOX gas sensors three of these sensors were prepared with the RGTO thin film technology and the other three constructed with nanowire technology. In fact, two of the three nanowires in the array are zinc oxides sensor but with different operating temperatures and the third one is a tin oxide sensor.

**Figure 16:**
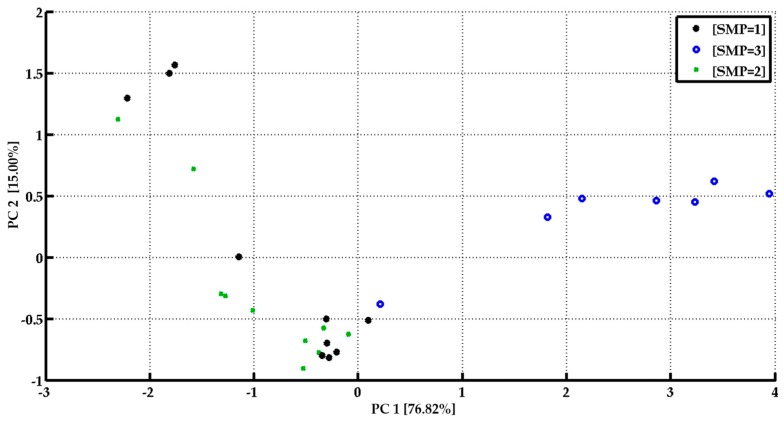
PCA score pot of the analysis carried out on samples belonging the three different individuals’ microbiota.

Figure 16 shows the Principal Component Analysis (PCA) resulting from the measurements carried out with samples collected from the 3 different individuals. The PC1 contain the 76.82% of the explained variance while the PC2 contains the 15.00%. It is possible to see that samples belonging to samples 1 and 2 are overlapped on the left side of the PCA while sample 2 follow a trend along the PC1. The position of the sample 2 respecting the PC1 axis is determined by the analysis time suggesting a progressive change in the head-space of the sample.

These results have been further supported by GC-MS-SPME analysis. Samples 1 and 2 showed similar head spaces and share some compounds with the sample 3. Common acids like acids of short chain as hexanoic, heptanoic, octanoic acid and alcohols such as 2-Nonadecanol, are used in the glucidic metabolisms of bacteria. Regarding the aldehydes group in HS, nonanal and octadecanal that aldehydes present only in sample 3. One of the most important compounds that is present just in sample 2 is indole and its concentration increases with time. It can be produced by a variety of bacteria through the metabolism of the amino acid tryptophan, being widely distributed in the natural environment.

### 3.3. Bacterial Contamination of Vegetable Soups

Thinking to the food industry, the electronic nose could be a useful tool in the production line, both to assist in food processing such as bread-baking [116], eventual transfer of plastic aromas from food plastic packages [117] as well as microbial contamination. Particularly for this last application, the existing techniques used in laboratories for the microbial screening in foods are techniques that require a long analysis time or massive costs to complete the tests. The established methodologies to deal with this challenge are diverse and range from microbiological analysis to sensory test panels to classical analytical approaches. Recent works have demonstrated the possibility to employ ENs in various food contexts such as process monitoring, freshness evaluation, shelf-life investigation, authenticity determination and product traceability and to diagnose microbial contamination in various food products. In particular, ENs based on metal oxide gas-sensors are reported as effective tools for microbial screening purposes in different types of food, including meat, fruits, grains, milk, providing a correct classification of samples with a score exceeding 85% in most of situations [118]. Beside this remarkable score, different review papers highlights how the capability to enhance the selectivity of sensors/ENs is one of the key issues to further progress in the performances of such devices [118,119]. For this purpose, sampling methods based on solid-phase microextraction (SPME), have been successfully applied, but at the cost of increasing the system complexity and response time [119]. Response time, in particular, is a key factor to successfully integrate EN systems in the production chain of food industries.

Indeed, a fast, on-line, identification of microbiological contamination, will save huge product, process and economic losses for the company. In this context, the work carried out at SENSOR lab in collaboration with Consorzio Casalasco del Pomodoro to detect bacterial (*Enterobacter hormaechei*) contamination in vegetable soups is reviewed as a remarkable example [98,120,121].

For these experiments we used a vegetable soup that is a mix of vegetable (carrots, potatoes, onions, leeks, broccoli, celery, grean beans) in which was inoculated a monoculture of *Enterobacter hormaechei*. Different concentration of microrganism were inoculated in to the food matrix and left to incubated at 35 °C for different time session (3, 5, 7, 12, 15, 17 and 24 h) in order to identify the best detection time. All samples were aliquoted in vials, closed and left 3 h to created the headspace. The experiments were carried out with the EOS507C electronic nose coupled with a dynamic headspace autosampler [122,123]. The EOS507 was equipped with an array of six metal oxide semiconductor (MOX) sensors and implemented with functionalities such as real-time sample humidity compensation, sensor response linearization and automated periodic calibration [98].

**Figure 17:**
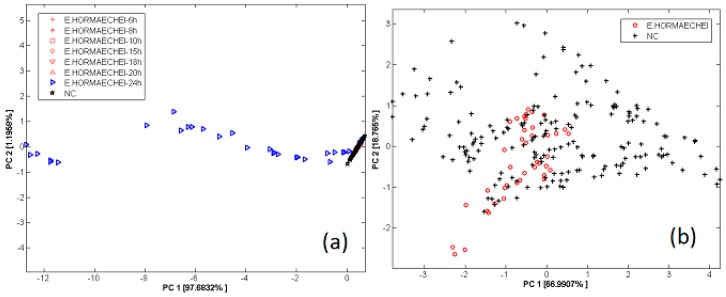
(**a**) PCA score plot of EN patters of samples not contaminated (NC) and contaminated by *E. hormaechei* detected in 24 h; (**b**) PCA score plot of EN patters of samples not contaminated (NC) and contaminated by *E. hormaechei* incubated for 6 h to 20 h (zoom of previous PCA reported in Figure (**a**)). Reprint from [120], copyright (2014) with permission from Elsevier.

The obtained results are shown in Figure 17, showing the suitability of the electronic nose to discriminate the contamination since 24 h of incubation. All contaminated samples incubated less than 24 h, are located inside the cluster of not contaminated samples in the right part of Figure 10b.

**Figure 18:**
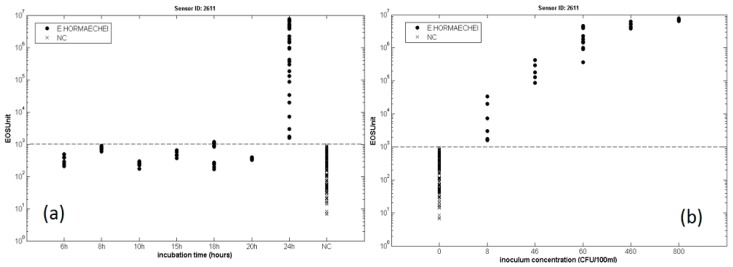
(**a**) plot of the sensor 2611 EOS units against the sample incubation time from 6 h to 24 h. C and NC samples are labelled in different ways (dots vs. crosses); (**b**) plot of the sensor 2611 EOS units as a function of the inoculum concentration (only C samples incubated for 24 h). Reprint from [120], copyright (2014) with permission from Elsevier.

The large range of electronic nose patterns of contaminated samples depends on the different initial concentration (as better discussed in Figure 18), which, in turn, causes a different final concentration as a consequence of the microorganism growth. The classification of bacterial contamination was performed using 5-fold CROSS Validation Linear Discriminant Analysis (5CV-LDA), with a performance of classification of 98.8%.

The effect of the initial concentration is better explained in Figure 18, which reports the response obtained with one sample sensor present inside the electronic nose. In Figure 18b, all the samples contaminated for a time of 24 h can be correctly classified with the minimum detection threshold around 10 CFU/100 mL, which is compatible with typical contamination values that may occur during actual production. Figure 18a further shows that the samples incubated less than 24 h are not classified as contaminated samples since their signals are close to the threshold line of detection. In parallel with the analysis made by electronic nose, the microbiological counting was also used to confirm the EN observations. The analysis of these results leads to the conclusion that the electronic nose is an excellent instrument for microbiological screening, but is important to considerer that the diagnosis is limited by the production of volatile metabolites that can occur after few hours of growth. For these reasons, the electronic nose could be a useful tool for food companies since it would allow to lower the time of detection and thus to reduce the costs.

## 4. Conclusions

In this special issue dedicated to the state of the art of sensor technology in Italy, we have reviewed results reached in the last years at the SENSOR laboratory, Brescia (Italy) focusing on selectivity as one of the major topics in metal oxide gas sensors.

In addition to the widely used conductometric device, devices based on other sensing mechanisms, namely surface ionization, photoluminescence and magnetic (magneto-optical Kerr effect) have been studied and are further being studied with the aim to develop alternative and complementary approaches to gas sensing. These results would be implemented in the future into a single device featuring a multi-parametric readout for selective sensing, offering a potential alternative to the traditional electronic nose solution.

Beside these activities, the more consolidated electronic nose approach has also been studied, mainly as a tool to detect microbiota activity in different media. The complexity of the headspace developed by such biological systems make the electronic nose a particularly appealing approach. Two key examples have been here reviewed showing the suitability of the EN to track these phenomena in different context: microbiota growth in human sweat, chosen due to its impact in different fields ranging from health to medicine and security; bacterial contamination of vegetable soups, chosen as a remarkable application for the food market.
